# Supplementary material for: Using blood test parameters to define biological age among older adults: association with morbidity and mortality independent of chronological age validated in two separate birth cohorts
Source: GeroScience. 2022 Sep 24;44(6):2685–99. doi: 10.1007/s11357-022-00662-9 (PMC9768057; doi:10.1007/s11357-022-00662-9)
Supplement: Supplementary file 1 — Supplementary file1 (DOCX 32.6 KB) [file 11357_2022_662_MOESM1_ESM.docx]

**Supplementary Material**

Table A.1
*Hazard Ratios for Mortality by Variables Defining Biological Age in the Berlin Aging Study*

|  | Hazard Ratio | [95% CI] | *N* |
| --- | --- | --- | --- |
| Zinc | 0.858* | 0.779–0.945 | 489 |
| Sodium | 0.857* | 0.781–0.941 | 505 |
| Chloride | 0.902* | 0.824–0.986 | 502 |
| Uric acid | 1.235* | 1.120–1.363 | 504 |
| Albumin | 0.893* | 0.819–0.974 | 505 |
| Alpha1 globulin | 1.139* | 1.050–1.236 | 498 |
| Alpha2 globulin | 1.138* | 1.037–1.250 | 498 |
| HbA1c | 1.243* | 1.124–1.376 | 451 |
| Hemoglobin | 0.856* | 0.765–0.959 | 494 |
| Leukocytes | 1.792* | 1.374–2.336 | 494 |
| Lymphocytes | 0.543* | 0.410–0.720 | 477 |
| Creatinine normed | 0.843* | 0.735–0.967 | 507 |

*Note*. Each row reports hazard ratios from separate models that included a given marker (z-standardized) along with chronological age, sex, and education. The only exceptions are coefficients for leukocytes and lymphocytes that were obtained from a conjoint model including both markers (in univariate analyses, the predictive effects of lymphocytes did not reach conventional levels of statistical significance). For parsimony, the predictive effects of chronological age, sex, and SES are not reported.

* *p* < .05.

Table A.2
*Hazard Ratios for Mortality by Variables not used to define Biological Age in the Berlin Aging Study*

|  | Hazard Ratio | [95% CI] | *N* |
| --- | --- | --- | --- |
| Lipase | 0.999 | 0.909–1.099 | 502 |
| Potassium | 1.012 | 0.920–1.112 | 504 |
| Calcium | 0.953 | 0.865–1.050 | 503 |
| Phosphate | 1.015 | 0.922–1.119 | 503 |
| Protein | 0.955 | 0.872–1.045 | 505 |
| Bilirubin | 0.982 | 0.884–1.090 | 504 |
| Cholesterol | 0.943 | 0.853–1.042 | 505 |
| SGOT | 0.956 | 0.867–1.054 | 506 |
| SGPT | 0.962 | 0.871–1.062 | 506 |
| SGGT | 1.038 | 0.975–1.105 | 506 |
| Alkaline Phosphatase | 1.017 | 0.934–1.107 | 507 |
| Beta globulin | 0.981 | 0.900–1.069 | 498 |
| Gamma globulin | 1.073 | 0.980–1.176 | 498 |
| Erythrocytes | 0.911 | 0.813–1.021 | 494 |
| Mean Corpuscular Hemoglobin | 0.939 | 0.844–1.044 | 494 |
| Mean Corpuscular Hemoglobin Concentration | 0.898 | 0.809–0.997 | 494 |
| Mean Corpuscular Volume | 0.989 | 0.889–1.099 | 494 |
| Thrombocytes | 1.075 | 0.972–1.189 | 493 |
| Quick | 0.958 | 0.872–1.052 | 489 |
| PTT | 0.975 | 0.900–1.056 | 487 |
| Fibrinogen | 1.087 | 0.996–1.187 | 468 |

*Note*. Each row reports hazard ratios from separate models that included a given marker (z-standardized) along with chronological age, sex, and education. For parsimony, the predictive effects of chronological age, sex, and SES are not reported.

* *p* < .05.

Table A.3
*Testing the unique predictive effects of biological age vis-a-vis those of chronological age, sex, and SES.*

| Predictors | % explained variation | *Δ* % explained variation (unique variance) |
| --- | --- | --- |
| Chronological age, women, SES, biological age | 26.81 | – |
| ~~Chronological age,~~ women, SES, biological age | 18.60 | 8.21 |
| Chronological age, ~~women~~, SES, biological age | 26.04 | 0.77 |
| Chronological age, women, ~~SES~~, biological age | 26.79 | 0.02 |
| Chronological age, women, SES, ~~biological age~~ | 24.64 | 2.17 |

Table A.4
*Intercorrelations among the Alternative Age Markers in the Berlin Aging Study II*

|  | Intercorrelations | | | | | |
| --- | --- | --- | --- | --- | --- | --- |
|  | 1 | 2 | 3 | 4 | 5 | 6 |
| (1) Telomere length (0.17–1.93) | 1 | –.01 | .01 | –.02 | .00 | .10* |
| (2) DNA methylation age – chronological age (–22.93–26.61) | –.01 | 1 | .23* | .03 | .14* | .14* |
| (3) Biological age – chronological age (–28.52–22.77) | .02 | .04 | 1 | .10* | .19* | .39* |
| (4) Skin age (0–3) | –.02 | –.04 | .02 | 1 | .09* | –.08 |
| (5) Proportional subjective age (–0.66–0.15) | .05 | –.04 | –.04 | .01 | 1 | –.04 |
| (6) PhenoAge – chronological age (–16.60–29.76) | .12* | .17* | .36* | –.04 | –.03 | 1 |
| *N* | 1,460 | 1,395 | 1,517 | 1,240 | 1,382 | 1,217 |
| *M* | 1.14 | 0.02 | 0 | 1.72 | –0.12 | –0.44 |
| *SD* | 0.23 | 6.91 | 6.10 | 0.90 | 0.09 | 5.29 |

*Note*. Raw intercorrelations between telomere length, DNA methylation age, biological age, skin age, subjective age, and PhenoAge are reported above the diagonal, intercorrelations that are residualized for age are reported below the diagonal.
**p* < .05.

Table A.5

*Predicting Physician-Observed Morbidity, Lung Capacity, and Subjective Health from Five Different Alternative Age Indice in BASE-II excluding PhenoAge: Shared and Unique Effects*.

| Predictors | Model 1 | Model 2 | Model 3 | Model 4 | Model 5 | Conjoint model |
| --- | --- | --- | --- | --- | --- | --- |
| Physician-observed morbidity | | | | | | |
| Chronological age | .007 | .002 | –.039 | .066* | –.009 | –.064 |
| Women | –.068* | –.053 | –.069* | –.058 | –.034 | –.076 |
| Education | –.018 | –.030 | –.032 | –.029 | –.002 | .014 |
| Telomere length | –.029 | –– | –– | –– | –– | –.001 |
| DNA meth^1^ | –– | –.058 | –– | –– | –– | .040 |
| **Biological age** | **––** | **––** | **.145*** | **––** | **––** | **.144*** |
| Skin age | –– | –– | –– | –.014 | –– | .029 |
| Subjective age | –– | –– | –– | –– | –.048 | .032 |
| Total *R^2^* | .005 | .008 | .023 | .009 | .003 | .028 |
| *F* | 1.35 | 2.31 | 7.07* | 2.30 | 0.89 | 2.50* |
| (*df1*, *df2*) | (4, 1,164) | (4, 1,172) | (4, 1,205) | (4, 987) | (4, 1,127) | (8, 687) |
|  | | | | | | |
| Lung capacity | | | | | | |
| Chronological age | –.210* | –.220* | –.191* | –.220* | –.223* | –.212* |
| Women | –.710* | –.704* | –.709* | –.715* | –.715* | –.743* |
| Education | .033 | .044 | .047 | .050 | .021 | .039 |
| Telomere length | –.004 | –– | –– | –– | –– | –.032 |
| DNA meth^1^ | –– | .003 | –– | –– | –– | –.045 |
| **Biological age** | **––** | **––** | **–.023** | **––** | **––** | **.004** |
| Skin age | –– | –– | –– | .005 | –– | .010 |
| Subjective age | –– | –– | –– | –– | –.018 | .030 |
| Total *R^2^* | .544 | .548 | .556 | .554 | .556 | .559 |
| *F* | 175.34* | 179.66* | 188.46* | 144.54* | 179.58* | 53.60* |
| (*df1*, *df2*) | (4, 587) | (4, 594) | (4, 603) | (4, 466) | (4, 573) | (8, 339) |
|  | | | | | | |
| Subjective health | | | | | | |
| Chronological age | –.025 | –.026 | –.034 | –.063* | .137* | .187* |
| Women | –.090* | –.086* | –.055* | –.033 | –.072* | –.048 |
| Education | –.000 | –.020 | .014 | .022 | –.009 | .019 |
| Telomere length | –.019 | –– | –– | –– | –– | –.053 |
| DNA meth^1^ | –– | .001 | –– | –– | –– | –.003 |
| **Biological age** | **––** | **––** | **–.101*** | **––** | **––** | **–.099*** |
| Skin age | –– | –– | –– | .076* | –– | .039 |
| Subjective age | –– | –– | –– | –– | –.289* | –.283* |
| Total *R^2^* | .008 | .008 | .011 | .009 | .066 | .066 |
| *F* | 2.42* | 2.41* | 3.72* | 2.47* | 21.59* | 6.62* |
| (*df1*, *df2*) | (4, 1,107) | (4, 1,111) | (4, 1,125) | (4, 893) | (4, 1,060) | (8, 645) |
|  |  |  |  |  |  |  |

*Note*. DNA meth = DNA methylation age. standardized prediction effects (*β*). ^a^ *p* = .07, ^b^ *p* = .05, * *p* < .05.

Table A.6

*Predicting Physician-Observed Morbidity, Lung Capacity, and Subjective Health from each of the residualized age indicators (by age) separately to examine the unique contribution of each age indicator*

| Individual Predictors | Physician-observed  morbidity | | Lung capacity | | Subjective health | |
| --- | --- | --- | --- | --- | --- | --- |
|  | Stand. Beta | R^2^ | Stand. Beta | R^2^ | Stand. Beta | R^2^ |
| Telomere length^R^ | .004 | .000 | –.166* | .028 | –.001 | .000 |
| DNA meth^R^ | –.009 | .000 | –.195* | .038 | –.006 | .000 |
| PhenoAge^R^ | .222* | .049 | .139* | .019 | –.102* | .010 |
| Biological age^R^ | .119* | .014 | –.070 | .005 | –.098* | .010 |
| Skin age^R^ | .004* | .061 | –.145* | .021 | –.043 | .002 |
| Subjective age^R^ | .012 | .000 | –.186* | .035 | .010 | .000 |

Note: ^R^=z-standardized residual values. DNA meth = DNA methylation age
